# Medical student perspective on stress: tackling the problem at the root

**DOI:** 10.1080/10872981.2019.1633173

**Published:** 2019-06-17

**Authors:** Amirali Fernandes, Ruhi Shah, Sparsh Shah

**Affiliations:** GKT School of Medicine, King’s College London, London, UK; Barts and The London School of Medicine and Dentistry, London, UK

Dear Editor,

Bergmann et al. provide invaluable perspective on the burden of academic and non-academic stress for medical students in Germany [1]. As final-year students in London, we have seen our medical programme evolve with time, to better combat the ever-growing problem of medical student stress. We believe our experiences may add useful insight to this global issue.

Understanding the complex interplay between academic and social stress is vital to ‘inform potential interventions,’ [1]. Bergmann et al. suggest introducing compulsory stress management training to the curriculum. Whilst this has merit in reducing perceived stress, we believe it fails to tackle the root of the problem [2]. Furthermore, mandatory additions to syllabuses may simply compound existing student time conflicts. Medicine is a continually expanding subject, with increasing expectations of student knowledge and competencies. We share examples of how system-level changes, rather than coping strategies, can mitigate growing demands, and reduce resultant student stress [3].

To avoid medicine becoming a life-consuming domain as the authors describe, our university offers ‘Wellness Weeks’ [1]. These encompass activities like yoga, cycling, dog-therapy and smoothie bars, free of cost and across all campuses, reducing any time-based or financial conflicts. Additionally, most universities nationally dedicate Wednesday afternoons to student participation in sports and hobbies. This university-supported, designated free time, combats the guilt associated with extra-curricular activity described by Bergmann et al. [1].

We resonate strongly with the described fear of examination failure and expulsion from medical school [1]. Whilst Bergmann et al. propose reducing examination grading to a pass-fail system, we found recent introduction of progress testing at our University allowed robust and valuable feedback on our performance over time. Through sitting multiple formative tests at the level of our final examinations, we began to see assessments as steps on a pathway of meaningful progress rather than intimidating terminal examinations [4]. The introduction of ‘Deans and Doughnuts’ mornings, where students can openly discuss fears, suggestions or simply have a conversation with senior faculty, has promoted student voice and value, creating a closer-knit community. Such activities bridge the gap between students and faculty, reducing fear created by perceived hierarchy.

Finally, our student-led ‘Mums and Dads’ programme promotes mentorship for first-year medical students by senior students, as we recognise the ‘beginning is a particularly stressful stage’ of medical school [1]. Peer-to-peer sharing of experiences through such programmes has been shown to promote cohesion and reassurance to junior students that they too can progress and succeed [5].

We strongly believe the continued sharing of ideas and innovation within the medical education community is vital for promoting student mental and physical wellbeing. It is only by looking after today’s medical students that we ensure a healthier future for tomorrow’s doctors.
